# The evolution of withdrawal: negotiating research relationships in biobanking

**DOI:** 10.1186/s40504-014-0016-5

**Published:** 2014-10-05

**Authors:** Karen Melham, Linda Briceno Moraia, Colin Mitchell, Michael Morrison, Harriet Teare, Jane Kaye

**Affiliations:** Department of Population Health, University of Oxford, Oxford, OX3 7LF UK

**Keywords:** Biobank, Right to withdraw, Research ethics, Consent to governance

## Abstract

The right to withdraw from research, along with the necessity of adequately informed consent, is at the heart of the post-Nuremburg code of ethical safeguards in biomedical research on human participants. As biomedical research moves away from direct interventional studies towards research using networks of linked human tissue samples and data, however, questions arise about what withdrawal can and should mean in these new contexts. Some of the more expansive traditional understandings, such as the right to withdraw from a study ‘at any time’ are limited in practice by the nature of biobank- supported research, particularly where it makes possible widespread dissemination and ongoing reuse of data. It is time for a more nuanced, granular arrangement for withdrawal, appropriate to the ongoing relationships between participants and long-term biobanking enterprises.

## Introduction

The right to withdraw is a central tenet of medical research ethics. It protects the autonomy of participants (Gertz 2008) and indeed, the option for an individual to withdraw is a measure of whether participation is voluntary (Wertheimer 1996). As such, the right to withdraw has helped to frame the relationship between researcher and participant. The principles of research ethics originated from the Nuremburg Trials and were initially articulated in relation to a particular kind of research, which was characterised by physical intervention. However, in recent years there has been a shift away from ‘traditional’ biomedical research on the physical bodies of individuals, to studies involving networks of data and collections of human tissue (Lipworth et al. 2011). This new kind of research is characterised by the development of biobanks: sustainable infrastructures supporting multiple, open-ended research projects. Biobanks entail the collection of large numbers of tissue samples combined with detailed medical and lifestyle data from patient participants or healthy volunteers. The need for ever larger sample sizes is driven by the promise of genetic and genomic epidemiology to understand the biological and environmental components of common complex diseases (Master et al. 2012; Smith and Aufox 2013). Technological innovation has concomitantly increased the ability to generate and disseminate large quantities of information, such as sequencing data, creating international networks of linked, shared research data (Kaye 2011). The risks associated with these new ways of carrying out research have less to do with direct physical harm than with disclosure of sensitive personal information. As a result, it has been argued that the nature of the relationship between research scientists and research participants is also changing (Meslin and Cho 2010; Kaye et al. 2012, although see also Corrigan and Tutton 2006).

Despite these developments, the meaning and practice of the right to withdraw from research has remained relatively unexamined (Holm 2011, Edwards 2005, Schaefer and Wertheimer 2010, Helgesson and Johnsson 2005). While participation and withdrawal may seem to be straightforward in projects involving procedures or interactions between researchers and individuals, it is much less clear how they might operate in practice in this new world of medical research that deals with stored samples, secondary research, and ‘big data’ approaches. Taking this gap in the literature as its starting point, this paper sets out to explore the concept of withdrawal from research and to document how this provision to protect voluntary participation in research has developed over time. We then consider in more detail how the nature of biobanks affects the meaningful possibilities for withdrawal and to what extent this is reflected in practice in the withdrawal policies of several major population biobanks. Returning to the relationship between researchers, biobanks and participants, we suggest that a more nuanced and limited form of withdrawal better expresses what is possible and desirable in the kind of long-term relationships on which these research infrastructures rely. The paper describes both the possibilities and limits of withdrawal and, through recognition of the evolution of both withdrawal and research, suggests how understandings of participation, research and withdrawal could be deepened and practice improved, for the benefit of both researchers and participants.

### The evolution of the right of withdrawal

The Nuremburg Code (1948) provides the original statement of the principle of voluntary informed consent for research, and of the concomitant right to withdraw from that research. This Code was developed in the aftermath of the Nuremburg Doctor Trials, in response to extreme and coerced human experimentation undertaken by Nazi doctors and researchers (Markman and Markman 2007). The focus of the Nuremburg Code is on invasive procedures; on interactions between researchers and participants that may challenge the physical integrity of participants. It was developed at a time when research ethics was in its infancy and information technologies were rudimentary.

It states:

“during the course of the experiment the human subject should be at liberty to bring the experiment to an end *if* he has reached the physical or mental state *where continuation of the experiment seems to him impossible*”^a^ (emphasis added).

It is striking how much more restrictive and conditional the right to withdraw is in this statement than in its later iterations and in current practice. This original understanding does not extend to the suggestions of later commentators that the right to withdraw is “unconditional and requires no explanation or justification” (Holm 2011). The Nuremburg code sets a very basic standard of conduct in response to atrocity, without reflecting in any depth on the nature of a research encounter.

Whereas the Nuremburg Code establishes provision for terminating an intolerable intervention in a research setting, the Declaration of Helsinki, the World Medical Association’s statement of principles for medical research, details further the right to withdraw by adding that there must be no reprisal for such termination. The Declaration has become the basis for many national statutes and is used as a guide by practitioners and research ethics committees around the world.^b^ In its 26^th^ principle, the Declaration (2013) states that

“…The subject should be informed of the right to abstain from participation in the study or to withdraw consent to participate at any time without reprisal…^c^”

The Declaration of Helsinki provides some theoretical justification for its position and places it in social context, noting that, “While the primary purpose of medical research is to generate new knowledge, this goal can never take precedence over the rights and interests of individual research subjects.”^d^ Further, the Declaration’s specification of a right to abstain or to withdraw *without reprisal* acknowledges the extent to which, particularly in medical research, an individual’s choices may be encumbered by clinical need. Because many participants are recruited by virtue of being patients, in order for their choice to be meaningfully voluntary there must be assurance that abstaining or withdrawing will not compromise their current and future clinical care. Safeguards such as voluntariness attempt to compensate for the natural vulnerability of potential participants (Chwang 2008).

The position of biomedical scientists is articulated by their representative body, The Council for International Organizations of Medical Sciences (CIOMS)^e^ in its “International Ethical Guidelines for Biomedical Research Involving Human Subjects” (2002).^f^ This document posits the centrality of informed consent as a measure that “protects the individual’s freedom of choice and respects the individual’s autonomy” and indicates that in order to be considered informed, a participant also needs to know that he or she “will be free to withdraw from research at any time without penalty or loss of benefits to which he or she would otherwise be entitled”. In this statement and in its reliance on traditional bioethical principles of respect for persons, beneficence, nonmalficence and justice (Beauchamp and Childress 2008), CIOMS aligns itself with the Declaration of Helsinki.

Both the Declaration of Helsinki and the CIOMS Guidelines also introduce – or rather, remove – a temporal element. Withdrawal, both positions assert, can be ‘at any time’. The wording is also repeated in the International Law through the Council of Europe Convention on Human Rights and Biomedicine^g^ and the European Union’s ‘Clinical Trials Directive’.^h^ This is a departure from Nuremburg’s provision to terminate an experiment that is proving physically or mentally intolerable. The right to withdrawal ‘at any time’ is different than the kind of immediate withdrawal brought about by cessation of an intervention. The introduction of this temporal element is an important factor in framing workable understandings of the right of withdrawal in non-interventional research.

Some international guidance also suggests that the right to withdrawal is much more extensive than may have been contemplated by the Nuremburg Code. The 2003 UNESCO International Declaration on Human Genetic Data sets out that, on withdrawal of consent to use of biological samples, “the data and biological samples should be dealt with in accordance with the wishes of the person”.^i^ This approach is echoed by CIOMS Guidelines for Biomedical Research, which state that “…subjects have the right to decide about such future use, to refuse storage, and to have the material destroyed^j^”.

An evolution of the right to withdraw to a point where it can be immediate, without giving reasons, and to the extent that all samples and data should be destroyed, is a dramatic extension of the conditional and minimal right originally set out in the Nuremburg Code. However, it may be that we have reached the high watermark of withdrawal.

The more recent CIOMS “International Guidelines for Ethical Review of Epidemiological Studies” (2009) consider the nature of consent in biobanks for the first time and, although not specific to biobanks, make recommendations for withdrawal in the context of epidemiological research. These guidelines recognise that in this form of research, withdrawal can present ‘special problems’^k^ and that it could take several forms. Withdrawal of consent to new data collection about the subject – the closest equivalent to cessation of an intervention – must be honoured. However, the guidelines leave open to researchers whether to fulfil requests for the removal of samples or data from storage altogether, and merely require that researchers state clearly at the outset if they do not intend to do so^l^.

Recognition of different forms of withdrawal and the limits imposed by the nature of certain kinds of research might represent a shift in approach, qualifying the scope of the right to withdraw and seeking to embed decisions about withdrawal in the context of reciprocal, ongoing relationships between participants and researchers/biobanks. In order to develop these ideas further, the nature and extent of the right of withdrawal will now be considered in light of the particular challenge of biobanks.

### The nature of participation in biobanks

The collection and storage of tissue and data for clinical research can be traced to the activities of hospital pathology labs such as the Karolinska Institutet^m^, the Icelandic Hospital Pathology collection,^n^ and the Austrian collections^o^ in the early twentieth century. While these pathology collections were relatively simple, contemporary biobanks exhibit considerable variation in scope, purpose, type of material collected, and participant population. Longitudinal biobanks begin with an initial collection of samples and data from individuals, which is then supplemented by further instances of data collection (and potentially additional sample collection), thereby building up a detailed biological, medical, lifestyle and environmental characterisation of the participant cohort as it develops over time. Population biobanks, such as the UK Biobank,^p^ tend to collect easily accessible samples such as blood or skin, accompanied by in-depth individual health and lifestyle information, from large cohorts of healthy volunteers for epidemiological and genomic research. Disease-specific biobanks focus on a particular condition or set of conditions, and collect disease-relevant tissue samples (e.g. tumour biopsies) and medical information from patients to support research into the underlying biology of the disorder of interest. Despite this heterogeneity, there are a number of conceptual and procedural challenges to post-war ethical standards for protecting human participants in biomedical research common to almost all biobanking enterprises.

One of the main reasons that the implementation of these ethical standards becomes problematic in the context of biobanking is that biobanking itself differs in substantive and important ways from the more traditional models of human subjects research that the Nuremberg Code and the Declaration of Helsinki were designed to govern. Whereas traditional biomedical research involves experiments conducted on a living person or persons, biobank participants undergo the process of donating their data and samples, but do not physically experience the research that is conducted using their material. Further, a biobank is not a research project *per se*; it is an infrastructure to facilitate research: it collects and curates blood, tissue and information obtained from individuals and makes this material available for researchers in an anonymised or coded (pseudonymised) form. Another key difference between studies based on physical interventions and biobank research is that it is inherent in the nature of a biobank that the use of the samples is not tied to delimited start and end times of a particular piece of research. Indeed, a biobank can be defined as “[a] collection of human tissue or other biological material, *which is stored for potential research use beyond the life of a specific project*” (emphasis added).^q^ Three questions then arise:

#### To what, exactly, does a biobank donor consent?

Consent to biobanking is qualitatively different from consent to an intervention or an experimental procedure. Biobank participants not only transfer their samples and data, they also transfer the right to make judgements about how the samples and data are used, with whom they can be shared, and for what ends. Since there is little or no direct interaction between the researchers studying the data and samples and the participants who donated them, the biobank (including its staff, policies and physical architecture) acts as an intermediary, mediating the flow of material between participant and researcher. Consent to biobanking is consent to this mediation, which is overseen according to the ‘rules of the game’ for that infrastructure, such as ethics committee review of access requests from external researchers, peer review and funder approval of research projects being necessary before researchers can access the biobank resource. We can conclude that: *Consent to biobanking is, in effect, consent to a regime of governance*.

#### What does withdrawal from a biobank mean?

With no risk of “direct physical harm” (Eriksson and Helgesson 2005) to the participant and open-ended research with no fixed end-point, the nature and meaning of withdrawal in biobanks is necessarily transformed*.* With regards to biological samples, there is some uncertainty as to what the implementation of the right to withdraw might entail; should withdrawal mean the samples should simply no longer be used for new research, or does it actively require the destruction of the samples as well? However, as identified by the 2009 CIOMS guidelines, it is in relation to the use of data that the greatest challenge of withdrawal from biobanking infrastructures lies. Data of the types commonly contained in biobanks, such as participant medical records, genetic or genomic sequences and sample characterisation metrics are routinely digitised, rendering them easily replicable, distributable and subject to modification, aggregation, and integration into larger data sets. Once data are shared with third parties outside the biobank it can be extremely difficult to trace, let alone control, what is done with a particular set of data derived from a specific research participant. It can be virtually impossible to ensure that a particular piece of data is removed entirely, especially if the data has been incorporated into a larger data summary where individual contributions can no longer be isolated. Similarly, data that has been published as part of an aggregated data set cannot meaningfully or practically be withdrawn at all from the public domain. In effect, past uses of data and samples cannot be undone and therefore withdrawal must by necessity focus on activities that have not yet begun, but that would otherwise be permitted under the normal ‘rules of the game’ of biobanking infrastructure. Therefore: *Rather than referring to cessation of an intervention, withdrawal now relates to prohibiting the future use of previously collected materials and data.*

#### What is the nature of the relationship between participant and biobank?

As we have seen, biobank participants have little or no direct relationship with researchers *per se*. Instead, the primary connection is between the participant and the biobank itself. This relationship, especially with population and longitudinal biobanks, is effectively open-ended. There is no fixed end point because there is no single fixed-duration research project. Consent – and being informed of the ‘rules of the game’ to which participants are agreeing – is part of the basis of this relationship, but it is not in itself sufficient. Successful long-term relations between participants and biobanks require trust (Watanabe et al. 2011), mutual engagement, and reciprocity (Hobbs et al. 2012). Both ethical and social perspectives and empirical research suggest that relations of trust and reciprocity require ongoing communication between biobanks and participants to match the ongoing nature of the research (Gottweis et al. 2011; Watanabe et al. 2011; Stein and Terry 2013). Gottweis and colleagues take this notion further, arguing that communication is important but not the only necessary component of this novel kind of relationship: “reciprocity is also a matter of building a culture of care for the study participants and transparency that is integral to a biobank. Such a culture is particularly important for study participants so that they gain a sense that they are making an important contribution to a project of high social relevance and that their act of giving is greatly appreciated” (Gottweis et al. 2011: 739). Members of a public deliberation on consent and withdrawal, for instance, asserted the need to balance individual autonomy with the public good of research and recognised the complexity of achieving that balance individually and as a society (Secko et al. 2009). Thus, *the nature of the relationship between participant and biobank needs to be one of partnership not power*.

Having established the contours of the changed dynamics of biobank research and participation compared to traditional interventional studies, we now return to the issue of withdrawal to examine how the right to withdraw is manifest in practice by looking at the withdrawal policies of several major population biobanks.

### Examples of withdrawal in the context of population biobanks

What the right to withdraw means for a biobank participant is articulated in a biobank’s consent form and the information sheet that accompanies it. The Public Population Project (P3G) based in Canada is an umbrella organisation that has developed many tools to harmonise biobanking activity.^r^ Resources include consent form and participant information templates that have been adopted by many biobanks around the world. What is meant by ‘withdrawal’ may be left undefined in a consent form. The P3G template consent form simply states: ‘I understand that my taking part is voluntary. I am free to withdraw at any time, without giving any reason and without affecting my present or future medical treatment.’ More information about what is meant by withdrawal is included in the template participant information sheet, which sets out options that a biobank may adopt:

“You are free to withdraw at any time from your participation in ______ and without giving any reason. …If you withdraw from the ______, your samples and the data derived from your sample and other personal information will be no longer used [Optional: destroyed]. If the data is already part of a dataset it cannot be destroyed. The code that enables us to re-link your samples and personal information will be deleted so that no further information about you will be collected”.

Or:

“Only your signed consent form and withdrawal will be kept as a record of your wishes. Such a withdrawal will prevent information about you from contributing to further research and analyses, but it will not be possible to remove your data from analyses that have already been done^s^”.

The materials developed by P3G can be adapted for use by various kinds of biobanks. For the purposes of clear comparison and illustration we have, in what follows, focussed on the withdrawal provisions of population biobanks only. These are examples of how withdrawal is handled in the practice of specific biobanks.

### CARTaGENE (Canada)

Participants in the CARTaGENE Population Biobank can withdraw ‘at any time’ and their data and biological samples will no longer be accessible to researchers.^t^ However, data and samples that have already been used cannot be withdrawn from current or completed studies: “As a participant, you are completely free to participate or not in the CARTaGENE project and you can end your participation at any time. …Data and biological samples from a participant that has withdrawn from the project will no longer be accessible to researchers via the CARTaGENE bank. However, data and samples that have already been used by researchers cannot be withdrawn from current or completed studies”^n^.

### Lifelines (Netherlands)

The Dutch biobank Lifelines gives participants the option to leave the study ‘at any time for any reason’ if they wish to do so, without any consequences. Participants will be asked to fill in a withdrawal form where they can choose whether or not LifeLines may continue to receive information about their health from their general practitioner and hospital and use this information for research purposes^u^.

### LifeGene (Sweden)

The Swedish LifeGene biobank provides for the three different levels of withdrawal: “no further contact”; “no further access”; and “no further use”. If a participant decides to withdraw, the biobank would seek written confirmation of the level of withdrawal the participant intends. LifeGene notes the limitations on withdrawal, stating that it

“will need to retain some minimal personal data for a number of reasons, which include: ensuring that participants who have withdrawn are not re-contacted; and assessing the determinants of withdrawal and any impact on research findings. Participants who withdraw will be assured that this administrative record will not be part of the main database that is available to others^v^”.

In cases where a participant has not actively withdrawn but may have been lost to follow up, LifeGene will continue to use the samples and data and maintain linkages, although it will not be able to update some data (e.g. those collected by repeat questionnaire).

### HUNT Biosciences (Norway)

This is one of two national biobanks in Norway, making available samples and data from the HUNT longitudinal population health studies. Participants can withdraw from the bank at any time and without giving a reason. In addition to describing what withdrawal entails, the Centre gives reasons for the practical limits to withdrawal: “The present practice at HUNT Research Centre is that withdrawal of consent will lead to the deletion of data and the destruction of the biological material. However, a withdrawal of consent will not have retroactive effect, i.e. ongoing projects keep the de-identified data and material. This practice provides a reliable framework for collaboration partners and guarantees the realization of projects without restrictions^w^”.

### UK Biobank

UK Biobank participant materials detail the different ways in which participants might withdraw from the biobank. Information about withdrawal is provided to potential participants in the Information Leaflet accompanying the initial invitation letter, under the heading ‘How do I withdraw if I want to do so?’. Participants are informed that they can withdraw at any time by telling members of staff via phone, email or online form. Participants are given three different levels of withdrawal: no further contact; no further access; and no further use. No further contact means that whilst participants would not be contacted again in the future, samples and information previously provided could continue to be used, together with information that would be obtained in the future from the medical record. No further access means that there would be no further contact, as well as no future access to information from the medical record, although information and samples previously provided would continue to be used. No further use means that, in addition to no further contact and no future information being obtained from the medical record, participant information and samples would not be available to researchers for future use, and samples would be destroyed. Participants are however notified that it might not be possible to trace all distributed sample remnants, that information would be retained for archival audit purposes, and that it would not be possible to remove information from analyses already undertaken^x^.

This is not intended to be a comprehensive review of withdrawal provisions found in consent and information forms, but some common elements emerge nonetheless. In all instances, it is made clear that participation is voluntary. Many forms also make reference to the fact that participants do not need to give a reason for withdrawal. However, what is meant by withdrawal may differ between projects, may be relatively undefined, and may not be explained to participants. Most biobanks appear to promise that information will not be used following the date of withdrawal, and that samples will be destroyed. Information already included in analyses will not – indeed, cannot – be removed. Once it forms part of completed research, it is necessary for purposes of research integrity and audit to maintain at least an archival record^y^.

Whether there can be levels of withdrawal or whether participation is ‘all or nothing’ largely depends on the operational capacity of the biobank to manage a variety of kinds of requests (Table 1).

**Table 1:** **Summary of the types of withdrawals in the biobanks analysed**

**Biobanks**	**Levels of withdrawal**	
	**Tiered**	**“All or nothing”**
**Cartagene Population Biobank** http://cartagene.qc.ca/en/documents		X
**Lifelines** https://www.lifelines.nl		X
**LifeGene** https://www.lifegene.se	X	
**HUNT Bioscience** http://www.ntnu.edu		X
**UK Biobank** http://www.ukbiobank.ac.uk	X	

### The evolution of withdrawal

The concept of withdrawal originated as a means of protecting human participants in biomedical research from being forced to undergo ‘unendurable’ physical experiences. Over time it has evolved to encompass a broader right to ‘walk away’ or decline to participate in research. It counters inherent imbalances in power between physicians and participants, many of whom are likely also to be patients. This evolution has given rise to the post-Nuremburg provisions that withdrawal can be ‘at any time’ and ‘for no reason’. However, as the focus of biomedical research has shifted away from direct physical interventions towards research using excised tissue samples and digitised health information, the meaning and relevance of these provisions is called into question. We are not arguing against the right to withdraw, but rather that this concept needs further consideration in order to be meaningful in biobanking contexts, and to fulfil the purpose for which it was developed. It is worth noting, for instance, that conditions of withdrawal cannot be applied to research that has already been completed and published. Nor can participants withdraw their samples and data from research projects that are currently underway. In effect, the right to withdraw refers to the right to prevent the future use of a participant’s material and data in new research projects. This understanding adds a degree of nuance to the concept of withdrawal ‘at any time’; a participant can indeed submit a request to withdraw from a biobank at any given point in time, but the nature of the practical implementation of this request means that the request will only affect future research. It is not the same as ‘walking away’ from a physical intervention study in which withdrawal and the effects of withdrawal are simultaneous.

There are practical limitations to what withdrawal from a biobank can entail, as outlined in the 2009 CIOMS guidelines. Ensuring that participants are aware, from the outset, of these limitations is part of properly informed consent. Another example of how withdrawal can be more nuanced is reflected in the ‘tiered’ withdrawal offered by population biobanks such as LifeGene and UK Biobank. Here, participants are effectively presented with different aspects of their relationship with the biobank from which it is possible to withdraw; communication (only), the ongoing collection of data (and no further communication) or ‘full’ withdrawal of their samples and data from future use in any new research. These different levels of withdrawal make explicit for the participants the different components of the participant-biobank relationship, and offer them more information on which to base their decisions about what it is they specifically want to withdraw from.

These are not the only options for making consent and withdrawal more informed. Whatever the level of withdrawal, the decision to do so is irreversible. A more participant-centred approach to research might recognise that in an ongoing study or research relationship such as a biobank, participants may want to be more engaged at some times than at others, or they may change their ideas about the kinds of feedback they want to receive and even the types of research they are happy to support. By moving from the ‘all or nothing’ approach to a nuanced selection of choices, participants can demonstrate active support for particular aspects of research by specifically consenting to those when agreeing to take part, and equally can withdraw from specific, limited, aspects of a biobank or research programme without having to leave the biobank altogether. A further step would be to create capacity for participants to be able to ‘reactivate’ earlier levels of participation if they wish. This type of granularity can potentially be achieved through mechanisms such as dynamic consent that use digital media tools to foster greater connection and reciprocity between (biobank) researchers and participants (Stein and Terry 2013; Wee 2013; Kaye et al. 2014).

Greater granularity and flexibility in consent arguably introduces a greater degree of empowerment, with participants being more directive in their support for research. From a logistical perspective, particularly in longitudinal studies that explicitly rely on reengagement with participants, it allows participants to take a break when other areas of their lives may need to take a greater priority or their circumstances change. Having the opportunity to reconsider and revisit previous decisions provides much greater flexibility, and control. This could be achieved using a dynamic consent interface by participants altering their communication settings via an online connection platform to the biobank in which they are enrolled.

This approach to withdrawal fits with the notion that consent should be considered to be the initiation of a partnership between researcher and participant, rather than a one-off decision at a single point in time. Providing greater choice for participants to determine how this relationship progresses is an important step in ensuring that the terms of this relationship are equal.

## Conclusion

The right to withdraw from medical research is central to medical research ethics. While this basic principle has not changed, the nature of some kinds of research has. Notions of withdrawal developed in relation to interventional research do not adequately address the kinds of research made possible by biobanks, nor the relationships that biobanks have with their participants. We argue not only that participants must be informed of the practical limits to withdrawal imposed by the structure of biobanks, but that articulation of different forms of withdrawal, and greater granularity and flexibility in the levels of both participation and withdrawal, encourage greater partnership between participants and researchers. We advocate a move to more nuanced, dynamic consent and withdrawal in place of blunt ‘all or nothing’ options for participation. This would enable understanding of the research relationship to be deepened and practice improved for the benefit of researchers, participants, and the research to which they are all committed.

### Endnotes

^a^Trials of War Criminals before the Nuremberg Military Tribunals under Control Council Law, 10 (2): 181. 1949. Washington, D.C.: U.S. Government Printing Office. http://history.nih.gov/research/downloads/nuremberg.pdf (accessed March 2014).

^b^In UK for instance, the Medicines for Human Use (Clinical Trials) Regulations 2004 refer explicitly to the ‘ethical principles that have their origin in the Declaration of Helsinki’ (Schedule 1, pt. 2, para. 1). http://www.legislation.gov.uk/uksi/2004/1031/contents/made (accessed March 2014).

^c^Declaration of Helsinki – Ethical Principles for Medical Research Involving Human Subjects. 1964. http://www.wma.net/en/30publications/10policies/b3/index.html (accessed March 2014).

^d^Declaration of Helsinki, General Principle 8.

^e^Council for International Organizations of Medical Sciences http://www.cioms.ch/index.php/about-us accessed Dec 2013 (accessed March 2014).

^f^International Ethical Guidelines for Biomedical Research Involving Human Subjects (CIOMS) 2002. http://www.cioms.ch/publications/layout_guide2002.pdf (accessed March 2014).

^g^Convention for the Protection of Human Rights and Dignity of the Human Being with regard to the Application of Biology and Medicine: Convention on Human Rights and Biomedicine (Oviedo Convention) 1997. http://conventions.coe.int/Treaty/en/Treaties/html/164.htm (accessed March 2014).

^h^Directive 2001/20/EC of the European Parliament and of the Council of 4 April 2001 on the approximation of the laws, regulations and administrative provisions of the Member States relating to the implementation of good clinical practice in the conduct of clinical trials on medicinal products for human use. http://eur-lex.europa.eu/LexUriServ/LexUriServ.do?uri=OJ:L:2001:121:0034:0044:en:PDF (accessed March 2014).

^i^UNESCO International Declaration on Human Genetic Data. 2003 http://portal.unesco.org/en/ev.php-URL_ID=17720&URL_DO=DO_TOPIC&URL_SECTION=201.html (accessed March 2014).

^j^International Ethical Guidelines for Biomedical Research Involving Human Subjects (CIOMS) 2002. http://www.cioms.ch/publications/layout_guide2002.pdf (accessed March 2014).

^k^International Ethical Guidelines for Epidemiological Studies (CIOMS) 2008. http://www.ufrgs.br/bioetica/cioms2008.pdf (accessed March 2014).

^l^Commentary on guideline 5, International Guidelines for Ethical Review of Epidemiological Studies (CIOMS, 2009).

^m^Karolinska Institutet http://ki.se/ki/jsp/polopoly.jsp?d=600&a=105529&l=en (accessed March 2014).

^n^Landspítali National University Hospital of Iceland http://www.landspitali.is/um-landspitala/languages/english/ (accessed March 2014).

^o^Medizinische Universität Graz http://www.meduni-graz.at/11984 (accessed March 2014).

^p^www.ukbiobank.ac.uk (accessed March 2014).

^q^Standard operating procedures for research ethics committees Version 5.1. 2012. http://www.hra.nhs.uk/resources/research-legislation-and-governance/standard-operating-procedures/ (accessed March 2014).

^r^Public Population Project in Genomics and Society http://www.p3g.org (accessed March 2014).

^s^Public Population Project in Genomics and Society http://www.p3g.org/resources/biobank-toolkit (accessed March 2014).

^t^CARTaGENE http://cartagene.qc.ca/en/documents (accessed March 2014).

^u^LifeLines Adult Protocol. 2013. https://www.lifelines.nl/lifelines-research/documents (accessed March 2014).

^v^LifeGene Ethics Policy. Version 4.3. 2010. https://www.lifegene.se (accessed March 2014).

^w^http://www.huntbiosciences.com/working-with-us/ethics-and-informed-consent (accessed March 2014).

^x^UK Biobank Ethics and Governance Framework, Version 3.0. 2007. https://www.ukbiobank.ac.uk/wp-content/uploads/2011/05/EGF20082.pdf?phpMyAdmin=trmKQlYdjjnQIgJ%2CfAzikMhEnx6Q24 (accessed March 2014).

^y^See, for instance, Guidance on Withdrawal of Subjects from: Data Retention and Other Related Issues – US Health and Human Services. http://www.hhs.gov/ohrp/policy/subjectwithdrawal.html (accessed March 2014).
